# The work-family interface and the COVID-19 pandemic: A systematic review

**DOI:** 10.3389/fpsyg.2022.914474

**Published:** 2022-08-04

**Authors:** Beatriz de Araújo Vitória, Maria Teresa Ribeiro, Vânia Sofia Carvalho

**Affiliations:** ^1^Center for Research in Psychological Science (CICPSI), Faculty of Psychology, University of Lisbon, Lisbon, Portugal; ^2^Faculty of Psychology, University of Coimbra, Coimbra, Portugal

**Keywords:** COVID-19, couples, parenting, work-family conflict, work-family enrichment, work-family balance, work-family boundaries, systematic review

## Abstract

In an unprecedented fashion, COVID-19 has impacted the work-family interface since March 2020. As one of the COVID-19 pandemic consequences, remote work became widely adopted. Furthermore, it is expected that other pandemics will occur in the future. Hence, this context represents a chance to gain deeper insight into telecommuters’ work and family spheres. Following PRISMA guidelines, the present narrative review aims to synthesise the COVID-19 impact on the work-family interface. Out of 121 screened references, 32 articles that measure at least one of the following variables–work-family conflict (25), work-family enrichment (3), work-family balance (8), and boundary management (21) were included. A thematic analysis using NVIVO12 was conducted, from which eight topics emerged: “paid workload, unpaid workload, and gender”; “well-being and gender”; “job resources, job demands, and gender”; “couples and gender”; “parenting and gender”; “occurrence of work-family enrichment with work-family conflict and gender”; “enforced blurred boundaries, its management, and gender”; “boundary management impact on work-family conflict, work-family enrichment, and work-family balance.” Overall, studies point out that COVID-19 had a complex effect on both work-family conflict and work-family balance, making it difficult to state whether these variables were mitigated or augmented. Findings demonstrated that COVID-19 produced little changes in work-family enrichment. As for the COVID-19 impact on work-family boundary management, individuals had to create new tactics to manage them due to the absence of boundaries between both systems. Besides, due to traditional gendered roles, the COVID-19 health crisis seems to have brought additional hurdles to couples and women.

**Systematic Review Registration:**

[https://www.crd.york.ac.uk/prospero/display_record.php?ID=CRD42021278254], identifier [CRD42021278254].

## Introduction

Since the 1930s (Frone, 2003), a growing body of research has addressed the work-family interface, mainly using three different lenses-conflict, enrichment, and boundary management (ten Brummelhuis and Bakker, 2012).

During the last decades, the work and family systems have been constantly metamorphosing due to the increasing number of dual-income couples (Vieira et al., 2014), women in the workforce, and technology use at work (Greenhaus and Powell, 2006). Nevertheless, the COVID-19 health crisis, without any kind of warning, has been challenging the work-family interface ever since March 2020. Indeed, the work system has been forced into families’ homes, vanishing all the pre-defined physical borders which delimited both systems (Shockley et al., 2021).

On January 31, 2020, the WHO declared a public health emergency due to the COVID-19 outbreak [World Health Organization (WHO), 2020b]. As the SARS virus quickly spread, on March 11, 2020, the WHO declared the COVID-19 world pandemic [World Health Organization (WHO), 2020a].

The different types of lockdowns (Anderson and Kelliher, 2020), the abrupt shift to mandatory remote work (Kramer and Kramer, 2020), closure of educational sites, imposition of online learning (Lian and Yoon, 2020), and the felt job insecurity (Blustein et al., 2020) were some of the challenges brought by the coronavirus disease. In this context, families, without any preparation, had to adjust to remote work, help their children adapt to online learning, and manage their children’s free time (Lian and Yoon, 2020). Additionally, families were interdicted to reach out to their social support network forced to share for the first time the same space 24/7 and to reshape all the relationships between the different systems and family subsystems (Shockley et al., 2021). All previous changes likely impacted the work-family interface (Lian and Yoon, 2020; Trougakos et al., 2020).

Of the challenges mentioned above, mandatory remote work is worthy of a closure gaze regarding its impact on the work-family interface. Telecommuting was introduced by Jack Nilles et al. (1976) as a work arrangement that could potentially decrease the amount of time spent commuting. It has been pointed out as a possible avenue for more flexible work schedules (Nilles et al., 1976). Working from home is a growing topic of interest among academics and organisations (Anderson and Kelliher, 2020). Notwithstanding, the increased interest in the subject does not mean remote work has become a widely applied practice. For example, in 2015, in Europe, on average, 27% of employees worked remotely (Eurofound and the International Labour Office, 2017). Teleworking has shown contradictory evidence in the work-family interface literature (Allen et al., 2015; Kelliher and de Menezes, 2019; Palumbo, 2020). Moreover, all empirical data on remote work was gathered when virtual working was chosen rather than imposed (Anderson and Kelliher, 2020).

The combination of imposed telecommuting with a reduced social support network appears to have boosted role strain (Thulin et al., 2019). Before coronavirus disease, wives were likelier to ensure unpaid work^1^ than their husbands (Craig and Powell, 2012). Moreover, in heterosexual relationships, men’s perception of women’s involvement in unpaid work tends to be more significant than the other way around (Cerrato and Cifre, 2018). During the 2020 lockdown, for example, according to Craig and Churchill (2020), dual-earners battled to manage the bulk of unpaid work. Furthermore, not only did women and men feel frustrated with managing paid and unpaid workload, but also wives were still more likely than their husbands to carry out domestic tasks (Craig and Churchill, 2020). Combined with the overall COVID-19 context, these links resemble work-family conflict (Greenhaus and Beutell, 1985), one of the main work-family interface frameworks (ten Brummelhuis and Bakker, 2012). Work-family conflict, a concept grounded on the role theory (Pleck, 1977), was defined as a form of inter-role conflict, which is felt when role demands are perceived as incompatible (Greenhaus and Beutell, 1985). Therefore, it is vital to understand if and how the coronavirus disease has altered work-family conflict.

Following the implications of combining enforced remote work with lockdown measures, nuclear families have spent more time together during the pandemic crisis than ever before (Lebow, 2020). The increased amount of time spent together could have changed the work-family interface. For instance, Nuru and Bruess (2021) and Weber et al. (2021) stated that most couples’ relationship quality was boosted or unaltered. These findings might be explained by individuals perceiving remote work as an arrangement that allows them to nurture their family relationships better. These findings evoke work-family enrichment, another widely used construct to study the synergies between work and family (ten Brummelhuis and Bakker, 2012). Based on the expansionist hypothesis (Sieber, 1974), work-family enrichment was defined as enhanced role performance resulting from perceiving gains and benefits in performing multiple roles (Carlson et al., 2006). Indeed, Powell theorises that work-family enrichment may have been boosted during lockdowns due to emotional and physical closeness between family members. However, whether or not this hypothesis is reflected in empirical findings remains unanswered (Powell, 2020).

It is paramount to understand work-family balance as an independent construct from work-family conflict and work-family enrichment (Carlson et al., 2009). Family closeness might have triggered changes in work-family balance. Work-family balance was conceptualised as meeting both role expectations, which were negotiated and shared between a person’s work and family responsibilities (Grzywacz and Carlson, 2007). Meeting both spheres’ obligations (work-family balance) might be shaped by plenty of other factors rather than work-family enrichment. Further, it might not only be prevented because of work-family conflict (Carlson et al., 2009). The above-mentioned family closeness could also align with the findings prior to the COVID-19 pandemic, which links spending quality time with family and job satisfaction (McNall et al., 2009). Furthermore, working from home (Virick et al., 2009; Lebow, 2020) and work-life balance (Chan et al., 2015; Noda, 2019) have been associated with family and life satisfaction. Finally, evidence on the relationship between remote work and work-family balance is somewhat conflicting (Allen et al., 2012) and biased once studied with employees who most likely have chosen this working arrangement (Anderson and Kelliher, 2020). Hence, it also seems essential to understand if and how the COVID-19 crisis has impacted work-family balance.

The blurring of boundaries between work and family systems resulted in family members being forced to share space (e.g., rooms) and objects (e.g., computers, tablets) so that parents could fulfil their work roles and children their student roles (Anderson and Kelliher, 2020). The new home arrangement of space and objects also played a crucial role so that family members could maintain connections with enlarged family members, friends, and the community (Lebow, 2020). Merging lockdowns with mandatory telecommuting yields the fourth main framework used to understand the interactions between the work and family systems: boundary management (ten Brummelhuis and Bakker, 2012). Built on a person-environment (P-E) fit theory (Harrison, 1978), boundary management assumes individuals create more flexible or rigid boundaries around work and family systems to navigate them (Ashforth et al., 2000; Kossek and Lautsch, 2012). Furthermore, individuals differ in their preferences to integrate or segment work and family (Edwards and Rothbard, 1999). The boundary theory body of research is based on the idea that physical and spatial boundaries segregate both systems (Ashforth et al., 2000). Such an assumption could not be further from the context offered by coronavirus disease. Additionally, amid the COVID-19 crisis, individuals were forced to blur boundaries between work and family, even if they had segmentation preferences. Therefore, the COVID-19 context offers a unique opportunity to understand how one adapts their boundary preferences to the family and which tactics individuals did use to navigate the two systems.

## The current study

On the one hand, other pandemics will likely occur during the 21st century (Frone, 2003), and remote work will become a standard job arrangement (Anderson and Kelliher, 2020). On the other hand, the work-family interface has been incipiently studied in times of crisis (Eby et al., 2016).

Since the COVID-19 pandemic is such a novel event, it is essential to synthesise its state of art. Hence, a narrative synthesis was the type of systematic review chosen because it allows drawing conclusions regardless of statistical significance and it also acknowledges a historical perspective (Siddaway et al., 2019) on the COVID-19 crisis. Moreover, a qualitative systematic review was likewise chosen due to the fact it enables comprehension of the COVID-19 impact on the work-family interface according to several different lenses (Morse, 2003). Hence, by synthesising mixed-method research, a systematic qualitative review enhances methodological rigour due to combining quantitative and qualitative research strengths while counterweighting their shortcomings (Johnson and Onwuegbuzie, 2004). Also, it eases the data access for decision-makers (Petticrew and Roberts, 2005).

Considering the SPIDER search tool (sample, phenomenon of interest, design, evaluation, research type), the present narrative systematic review was guided by questioning, “How has the COVID-19 pandemic impacted the work-family interface?”

Hence, and mindful of Trougakos et al. (2020), this systematic review synthesises how COVID-19 has impacted the work-family interface. Specifically, it seeks to (1) provide an overall view of the body of evidence regarding the COVID-19 impact on the work-family interface; (2) to map and compare the COVID-19 effects on the work-family interface on the marital subsystem, the parental subsystem, and the family system; to understand how the COVID-19 influenced (3) work-family conflict; (4) work-family enrichment; (5) work-family balance; (6) boundary management.

## Methods

The Preferred Reporting Items for Systematic Reviews (PRISMA) was used to guide the reporting of this review (Page et al., 2021). The current systematic review has been registered at PROSPERO (CRD42021278254).

### Eligibility criteria

#### Inclusion criteria

Studies published between December 2019 and March 2022 were included if the data was collected during the COVID-19 context. Moreover, all studies must have measured at least one of the work-family interface variables. Work-family variables were defined as work-family conflict, work-family enrichment, work-family balance, segmentation preferences, and integration preferences. Once the present systematic review focuses on the work-family interface, we included both studies with a dyadic or individual analysis if the sample was composed of employed individuals and/or couples (married, cohabiting, civil registration, and with or without children). The study design included qualitative, quantitative, and mixed studies. Because the COVID-19 pandemic is a novel event that has caught researchers’ attention, studies published in peer-reviewed academic journals and unpublished papers/early access and doctoral thesis were included. In addition, studies published in Portuguese, English, and Spanish were searched.

#### Exclusion criteria

All studies that solely measured the work-life interface or the work-non-work interface were excluded. Further, studies that included a sample with any prior condition (e.g., depression, anxiety, burnout, and multi-challenged families) were excluded to focus on the work-family interface framework. For the same reason, research aiming to understand the work-family interface in a particular professional sector (e.g., health workers, teachers, and social workers) were also excluded. Case studies, opinion articles, reports, or review articles were further excluded.

### Search strategy

With the advice of an external member from our research centre, a search strategy was developed in August 2021 and perfectioned until September 2021 (Supplementary Data Sheet 1). The following databases were included: EBSCO (Academic Search Complete, MEDLINE, APA PsycArticles, APA PsycInfo, Psychology, Behavioural Sciences Collection, ScienceDirect, ERIC, and RCAAP), Web of Science (Science Citation Index, Social Sciences Citation Index, Arts and Humanities Citation Index, Conference Proceedings Citation Index, and Emerging Sources Citation Index), Scopus and Google Scholar. Results were merged using Endnote Online by Clarivate for screening and extraction.

### Study selection and data extraction

The searches were run by BV and an external member from our department during October 2021. The two reviewers screened the titles and abstracts against the eligibility criteria. After, full-text documents were obtained for all articles considered possibly appropriate. After both researchers blindly extracted the final studies, a meeting was conducted to discuss differences. In this meeting, MR and VC were involved in the final selection of the articles, considering the inclusion and exclusion criteria. A final consensus was reached and agreed upon by all. To include more robust findings, between October and March 2022, BV updated the included articles by setting search alerts. The following data were extracted from the final set of included studies: author, date, sample type, study design, study analysis, hypothesis, and main results.

### Data synthesis

Firstly, a quality assessment of the final articles selected was conducted before analysing the main results (Harrison et al., 2021). Then, a qualitative synthesis was developed given the heterogeneity of study designs defined in the inclusion criteria (Popay et al., 2006) and the present systematic review aims.

### Quality assessment strategy

The extracted studies’ methodological quality was appraised using the revised version of the Quality Assessment for Diverse Studies tool (QuADS), developed by Harrison et al. (2021). QuADS has demonstrated consistent reliability and validity (Sirriyeh et al., 2011), and it was designed considering the context of psychology research, including mixed method studies (Harrison et al., 2021). Hence, and considering the inclusion criteria of the current systematic review, the QuADS tool was selected. The tool comprises 13 items measured using a 0–3 scale (Harrison et al., 2021). Consistent with the guidelines (Harrison et al., 2021), two reviewers (BV and VC) selected five studies and screened them with QuADS. Then, a meeting was scheduled to reach a shared understanding of using the tool. Due to time constraints, BV and VC selected 12 articles to screen independently. Afterward, an interrater analysis was carried out using the Kappa test, and a score of 0.298 was obtained (Gisev et al., 2013). Later, BV conducted the quality assessment for all the included articles. According to the literature (Harrison et al., 2021), no studies were excluded after the quality appraisal result.

## Results

### Search results

After exploring all the databases mentioned above, 32 articles were gathered. Considering the removal of the duplicates, 256 articles were screened against the information provided in the title and abstract. Next, 121 studies were obtained. Hence, following the PRISMA checklist (Page et al., 2021), the full text of these 121 articles was downloaded to screen them across the eligibility criteria. Email alerts in all databases were set to ensure we were up to date with the most recent studies. At the end of the screening and selection process, we found 21 studies that met the inclusion criteria. As a result of all the email alerts set, till March 2022, another 11 articles were added.

It is paramount to clarify that, during the data extraction, many borderline cases (Siddaway et al., 2019) were found regarding the sample’s inclusion criteria. Given the state of the literature, it was decided to include studies with different criteria for the professional sector, marital status, and the number of children. Regarding the professional sector, studies that collected data from a cluster of participants who worked in the same professional sector blended with professionals from other sectors were included. Papers whose sample was solely based on one professional sector were excluded. Following the same rationale, studies whose sample was heterogenous regarding marital status (e.g., single, cohabiting, civil partnership, divorced, widowed) were included; but papers whose sample were only single employees were excluded. Once more, articles composed of only participants who had never been parents were excluded; but papers whose sample criteria included with and without offspring were included. The researchers of the included studies were contacted by email or *via* ResearchGate when doubts arose. These doubts were mainly regarding the above-mentioned borderline cases or the exact timeline for gathering data.

**FIGURE 1:**
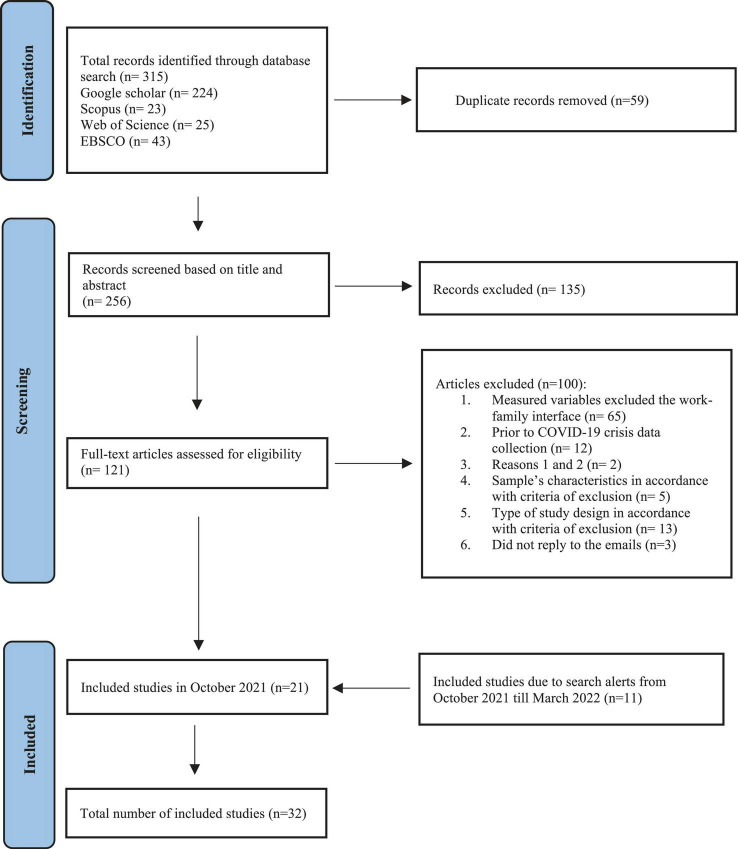
Summary of search results, adapted from PRISMA (Page et al., 2021).

The search process was meticulously and independently conducted by two researchers (BV and an external researcher from our department). The complete overview of this process can be found in Figure 1.

A thematic analysis was carried out using NVIVO12. Thematic analysis was chosen since it allows coding qualitative data and answering the research question by identifying patterns (Braun and Clarke, 2013). The thematic analysis went through several phases (Braun et al., 2019), being the first to get familiarised with the data by thoroughly reading the 32 articles. After, data segments were coded into coding nodes. Then, the process was revised so that central nodes, child nodes, and main themes could emerge. Finally, the emerging themes were discussed between BV, MR, and VC and crossed with the selected work-family variables.

Work-family conflict emerged in twenty-five articles, and it was crossed with five themes: “paid workload, unpaid workload, and gender,” “well-being,” “job resources, job demands, and gender,” “couples and gender,” and “parenting and gender.”

Work-family enrichment was measured in three articles, and it was interpreted considering three topics: “well-being and gender,” “job resources, job demands, and gender,” and “occurrence of work-family enrichment with work-family conflict and gender.”

Work-family balance was found in eight articles, and it was discussed in light of the following five themes: “paid workload and unpaid workload,” “well-being,” “job resources, job demands, and gender,” and “couples and gender,” and “parenting and gender.”

Boundary management emerged in twenty-one articles. It was dissected according to five topics: “enforced blurred boundaries, its management, and gender,” “well-being,” “job resources, job demands, and gender,” “couples, parents, and gender,” and “boundary management impact on work-family conflict, work-family enrichment, and work-family balance.”

### Articles excluded

In October 2021, at the full-text review, 100 studies were excluded. Sixty-five studies did not measure the work-family interface variables defined by the inclusion criteria. Twelve studies exclusively collected data before the pandemic, whereas two neither measured the work-family interface nor collected data during the COVID-19 crisis. Regarding the sample’s characteristics defined by the inclusion criteria, four studies focused on the challenges of a particular professional sector, one study on adoptive families. Lastly, concerning the research type, seven studies did not have an empirical nature (e.g., chapters, opinion articles), and six were master’s thesis. Doubts regarding three articles were emailed to the corresponding authors, but a reply was not obtained. For all the reasons mentioned above, these studies were excluded.

### Characteristics of included studies

In total, 32 articles were included. A summary of included studies and their characteristics can be found in Table 2 in Supplementary Data Sheet 2. Some studies collected data in more than one country. The present systematic review includes data from the United States (7), Portugal (4), United Kingdom (3), Netherlands (3), India (2), China (2), Turkey (2), Spain (2), Romania (2), Germany (2), Canada (2), Austria (1), Indonesia (1), Ireland (1), Poland (1), Singapore (1), Switzerland (1), Belgium (1), Australia (1), Bulgaria (1), Brazil (1), Japan (1), Malaysia (1), Nigeria (1), Ecuador (1), Israel (1), Serbia (1), and Finland (1). Most of the studies aimed to understand the experience of full-time workers in various sectors (16). In contrast, others focused on women’s perceptions (whether single, married, cohabiting, with or without children) (7), couples’ points of view (dual-earners, married, cohabiting, with or without children) (4), and working parents’ understanding (5). The majority of the studies adopted a cross-sectional design (29), with a few using a longitudinal design (2) or a quasi-experimental design (1). Two cross-sectional studies have collected data in two waves. The final selection of studies included quantitative data (18), qualitative data (9), and mixed-methods data (5). Besides this, only 1 study used a dyadic analysis. Out of the 32 articles, one is awaiting the peer review process, and one was provided through early access.

### Quality assessment results

While considering the present systematic review findings, it must be bear in mind the included studies revealed heterogeneity in its methodological quality. All the studies were developed in a strained time window since data collection was amid the COVID-19 crisis. We believe this could have impacted the research design and procedure quality. Further, once early access and not yet peer-reviewed articles were included, the manuscripts we were provided were not the final refined version. Both these facts were considered when performing the quality assessment. On the one hand, studies revealed fairly good theoretical underpinning, study design, method of analysis given the research aims, and discussion of strengths and limitations. On the other hand, studies showed compromising quality on the sampling approach to address the research aims, the description of the research setting and target population, and evidence that the research stakeholders were consulted for developing the research design or procedure.

## Findings

### Work-family conflict

#### Paid workload, unpaid workload, gender, and work-family conflict during the COVID-19 pandemic

Overall, paid workload strain was strongly incremented by the COVID-19 harsh imposed measures (Chung et al., 2020; Lemos et al., 2020; Wang et al., 2020; Adisa et al., 2021; Andrade and Fernandes, 2021; Andrade and Petiz Lousã, 2021; Aplin-Houtz et al., 2021; Asaari and Desa, 2021; Carvalho et al., 2021; Çetin et al., 2021; Chenji and Raghavendra, 2021; Čikić and Rajačić, 2021; Kolo et al., 2021; Leroy et al., 2021; Niu et al., 2021; Soubelet-Fagoaga et al., 2021; Stefanova et al., 2021; Zou et al., 2021). Moreover, employees had to adapt to enforce remote work at a fast pace (Chung et al., 2020; Wang et al., 2020; Andrade and Petiz Lousã, 2021; Aplin-Houtz et al., 2021; Barriga Medina et al., 2021; Carvalho et al., 2021; Chenji and Raghavendra, 2021; Čikić and Rajačić, 2021; Leroy et al., 2021; Niu et al., 2021; Soubelet-Fagoaga et al., 2021; Stefanova et al., 2021; Zou et al., 2021). Additionally, unpaid workload also increased (Chung et al., 2020; Wang et al., 2020; Aplin-Houtz et al., 2021; Carvalho et al., 2021; Chenji and Raghavendra, 2021; Čikić and Rajačić, 2021; Kolo et al., 2021; Leroy et al., 2021; Niu et al., 2021; Soubelet-Fagoaga et al., 2021; Stefanova et al., 2021; Zou et al., 2021).

All the variables mentioned above seem to have altered the work-family interface, resulting in increased role strain (Lemos et al., 2020; Adisa et al., 2021; Andrade and Fernandes, 2021; Andrade and Petiz Lousã, 2021; Aplin-Houtz et al., 2021; Asaari and Desa, 2021; Çetin et al., 2021; Çoban, 2021; Kolo et al., 2021; Soubelet-Fagoaga et al., 2021). However, if all these strains have led to an intensification of work-family conflict compared to before the COVID-19 health crisis is somewhat unclear.

On the one hand, some studies point out an overall work-family conflict growth during the COVID-19 outbreak compared to before, whether using a longitudinal design (Verweij et al., 2021), a two-wave cross-sectional design (Vaziri et al., 2020), or by using retrospective perceptions (Wang et al., 2020). On the other hand, teleworkers reported higher work-family conflict levels than commuters (Niu et al., 2021; Sedaroglu, 2021; Soubelet-Fagoaga et al., 2021) or than workers with a hybrid arrangement (Sedaroglu, 2021). In addition, findings show a link between work-family conflict magnification and enforced remote work (Andrade and Petiz Lousã, 2021; Kolo et al., 2021).

On the other hand, work-family conflict seems to have scored average in cross-sectional studies (Barriga Medina et al., 2021; Sedaroglu, 2021). These results could be in line with Vaziri et al. (2020), who conducted a two-wave study focused on the evolution of work-family conflict from February 2020 to April 2020. Regarding their findings related to work-family conflict, only a tiny percentage of individuals reported a fluctuation in work-family conflict (Vaziri et al., 2020). Taking a closer gaze at this small percentage of individuals, the most common transition was from low to medium levels of work-family conflict (Vaziri et al., 2020). The second most common transition was from medium to low levels of work-family conflict (Vaziri et al., 2020).

The couple studies that have measured work-to-family conflict and family-to-work conflict separately show that work-to-family conflict was higher than family-to-work conflict (Barriga Medina et al., 2021; Soubelet-Fagoaga et al., 2021). Increased family-to-work conflict in the COVID-19 crisis can be accounted for by unpaid workload intensification combined with having scarce access to support from educational facilities, household services, enlarged family members, and other community members (Aplin-Houtz et al., 2021; Kolo et al., 2021). Women frequently reported this scenario (Soubelet-Fagoaga et al., 2021).

Women who worked from home reported greater family-to-work conflict levels than men who worked remotely or women and men who commuted (Soubelet-Fagoaga et al., 2021). While complying with enforced remote work, work-family conflict was lower for women who were fond of saving time in commuting (Lemos et al., 2020; Čikić and Rajačić, 2021) and those who had their schedules reduced (Čikić and Rajačić, 2021). Indeed, in Čikić and Rajačić’s (2021) study, whose sample were females, solely 6.1% of women declared to be unable to manage work and family systems.

#### Well-being, work-family conflict, and gender during the COVID-19 pandemic

Work-family conflict was perceived as one of the enforced remote work challenges with a negative impact on emotional exhaustion (Wang et al., 2020) and distress due to the COVID-19 measures (Niu et al., 2021).

Work-family conflict (in both directions) meant higher levels of negative affect and lower levels of positive affect (Çoban, 2021). Additionally, the more time individuals spend at home, the stronger the relationship between work-family conflict and negative affect (Çoban, 2021).

A good core self-evaluation enabled individuals to better cope with work-to-family conflict; and enhanced individuals’ workplace well-being (Asaari and Desa, 2021).

During the COVID-19 health crisis, work-family conflict was linked to burnout (Sedaroglu, 2021). Cognitive weariness and emotional exhaustion were the most affected dimensions of burnout by work-family conflict (Sedaroglu, 2021). This seems to align with individuals reporting family-to-work conflict during the COVID-19 pandemic to be somewhat “chaotic,” adding strain to an already stressful situation *via* work exhaustion (Kolo et al., 2021; Waismel-Manor et al., 2021).

Working women, compared to working men, reported a higher impact on physical and mental symptoms (Čikić and Rajačić, 2021) and augmented burnout symptoms (Stefanova et al., 2021).

#### Job resources, job demands, gender, and work-family conflict during the COVID-19 pandemic

On the one hand, workload stressors (Wang et al., 2020; Chenji and Raghavendra, 2021; Čikić and Rajačić, 2021), high levels of work stress (Asaari and Desa, 2021; Sedaroglu, 2021), working after hours (Wang et al., 2020; Andrade and Petiz Lousã, 2021; Chenji and Raghavendra, 2021; Čikić and Rajačić, 2021), low job satisfaction (Vaziri et al., 2020), low job autonomy (Andrade and Petiz Lousã, 2021), reduced job performance (Vaziri et al., 2020; Chenji and Raghavendra, 2021; Čikić and Rajačić, 2021), increased economic pressure (Kolo et al., 2021), higher job insecurity (Barriga Medina et al., 2021; Čikić and Rajačić, 2021; Kolo et al., 2021), and more significant turnover intentions (Vaziri et al., 2020) were linked to work-family conflict during the COVID-19 crisis.

On the other hand, experiencing lower levels of work-family conflict was related to lower turnover intentions (Vaziri et al., 2020) and more robust job autonomy (Sedaroglu, 2021), job satisfaction, and job commitment (Vaziri et al., 2020).

Organisational (Asaari and Desa, 2021), co-workers (Wang et al., 2020; Chenji and Raghavendra, 2021), and supervisor support (Wang et al., 2020) as well as the preference for a flexible working schedule (Chenji and Raghavendra, 2021) mitigated work-family conflict amidst the COVID-19 outbreak. During the COVID-19 health crisis, support by supervisors and co-workers boosted work performance by shrinking family-to-work conflict; support by supervisors and co-workers lessened emotional exhaustion by mitigating work-to-family conflict; and support by supervisors and co-workers enhanced life satisfaction by lowering work-to-family conflict (Wang et al., 2020). Moreover, work-to-family conflict declined the well-being of employees with a more significant paid workload and intense job monitoring (Wang et al., 2020). Besides, supervisor support dampened the relationship between working after-hours and work-family conflict (Andrade and Petiz Lousã, 2021). Women perceived supervisor support as a critical factor restraining work-family conflict (Aplin-Houtz et al., 2021).

For women whose paid workload decreased during COVID-19, this fluctuation is also linked to lower job satisfaction since they felt pushed to gendered roles and feared future career setbacks (Çoban, 2021). Furthermore, work cessation was linked with feelings of job insecurity (Čikić and Rajačić, 2021). Additionally, job insecurity was associated with women reporting higher career aspirations during the lockdown than men (Stefanova et al., 2021). Moreover, while complying with enforced remote work, work-family conflict was lower for women who were discovering the benefits of having a more flexible working schedule (Lemos et al., 2020; Čikić and Rajačić, 2021) and for women who stopped working (Čikić and Rajačić, 2021). What’s more, women who were unable to manage both spheres not only struggled to organise work but also felt their job performance decreased (Čikić and Rajačić, 2021). Finally, for working women who were working from home, lower job performance was linked with organisational culture (Čikić and Rajačić, 2021).

#### Couples, gender, and work-family conflict during the COVID-19 pandemic

Individuals from dual earner-couples report high scores of work-to-family conflict (Efi and Parahyanti, 2021). Couples also shared that the switch to remote work was too fast (Kolo et al., 2021). The re-organisation of family members’ relationships and sharing of home space was another factor that boosted work-family conflict for couples (Kolo et al., 2021). Further, role strain felt during the COVID-19 outbreak made couples think there was no time to nurture their relationship since the professional and parent roles were more prominent than the spouse role (Kolo et al., 2021).

Couples felt the urge to manage work stress not to affect their marital and family relationship quality (Asaari and Desa, 2021). In addition, couples also aimed to prevent their family stress from negatively impacting their job (Asaari and Desa, 2021).

Apart from the work-family magnifiers, couples reported physical closeness and having more time together lowered work-family conflict (Kolo et al., 2021).

Women noted that their partners were more involved in unpaid work; nevertheless, they adopted the “assistant” role or had a poor performance (Chung et al., 2020; Çoban, 2021). Men’s paid and women’s unpaid work were prioritised (Soubelet-Fagoaga et al., 2021; Waismel-Manor et al., 2021). No significant differences were found between the number of hours dedicated to paid workload between men and women (Stefanova et al., 2021). On the contrary, women spent more time committed to unpaid workload (Stefanova et al., 2021). This cluster pushed women and men to perform traditional roles (Çoban, 2021; Soubelet-Fagoaga et al., 2021; Stefanova et al., 2021). Some results point out that work-family conflict was lower for women whose husbands shared unpaid workload (Čikić and Rajačić, 2021), while others point out that sharing the unpaid workload between spouses did not lessen work-family conflict (Lemos et al., 2020). Notwithstanding, among couples, women experience higher work-to-family and family-to-work conflict than men (Stefanova et al., 2021).

#### Parents, gender, and work-family conflict during the COVID-19 pandemic

One of the parents’ main struggles was assuming the educational role during the COVID-19 pandemic (Wang et al., 2020; Aplin-Houtz et al., 2021; Kolo et al., 2021). This might explain why family-to-work conflict among parents boosted depressive symptoms during the COVID-19 crisis, but not work-to-family conflict (Zou et al., 2021). This hurdle was related to unavailable outside the nuclear family support (Soubelet-Fagoaga et al., 2021). Indeed, employees with offspring reported higher work-to-family and family-to-work conflict levels than employees with no children (Soubelet-Fagoaga et al., 2021; Stefanova et al., 2021). Only one study, whose sample was working-married-Turkish-moms, showed that paid workload for these women decreased during the COVID-19 context, while paid workload for husbands increased or remained unaltered (Çoban, 2021).

Parents were mindful of managing their work stress not to affect their parental relationship (Çoban, 2021). Accordingly, the link between family-to-work conflict and work-to-family conflict with parenting remained unaltered compared to before the COVID-19 outbreak (Verweij et al., 2021).

It remains unclear if the COVID-19 outbreak has increased work-family conflict. Nonetheless, it becomes possible to answer this question by breaking it into four different groups–highly educated mothers, highly educated fathers, lower/middle educated mothers, and lower/middle educated fathers. Verweij et al. (2021), as well as Stefanova et al. (2021) findings, show that both work-to-family conflict and family-to-work conflict were greatly magnified across waves for mothers compared to fathers; and for highly educated parents than for medium/lower educated parents (Verweij et al., 2021). Comparing the four groups, the growth of work-to-family conflict and family-to-work conflict was more considerable for highly educated mothers, followed by medium/lower educated mothers (Verweij et al., 2021). Highly educated fathers scored the lowest increase in family-to-work conflict, whereas medium/lower educated fathers reported no fluctuations in family-to-work conflict (Verweij et al., 2021). Nonetheless, the case for work-to-family conflict is different since it only increased among highly educated mothers and significantly decreased for lower/medium educated fathers (Verweij et al., 2021). This surprising result is linked with lower/medium educated fathers’ spouses working fewer hours than the highly educated fathers (Verweij et al., 2021). Hence, it could be that the lower/medium educated fathers and their spouses have adopted a more gendered division of unpaid workload as work-to-family conflict declined for lower/medium educated fathers and increased for their spouses (Verweij et al., 2021).

Despite the role strain, work-family spilled very little for the parenting relationship. For mothers, work-family conflict slightly increased coerciveness, slightly decreased the perceived quality of the dyadic relationship, and had no effect on positive encouragement (Verweij et al., 2021). For fathers, results show no differences in all the three dimensions of the parenting relationship (Verweij et al., 2021).

Working mothers who reported increased paid and unpaid workload differed from the women who reported only one type of workload intensification since they had more children (Čikić and Rajačić, 2021). Compared to women who were not mothers, mothers spent less time dedicated to their job and more time dedicated to their children (Stefanova et al., 2021). Before the COVID-19 outbreak, mothers used to rely highly on their social support network to suppress their work-family conflict (Lemos et al., 2020; Aplin-Houtz et al., 2021; Çoban, 2021). Hence, due to the COVID-19-related measures, working mothers perceived higher work-family conflict during the COVID-19 health crisis compared to before (Andrade and Fernandes, 2021; Aplin-Houtz et al., 2021; Čikić and Rajačić, 2021; Çoban, 2021; Verweij et al., 2021). Tangibly for working mothers, the role strain (Lemos et al., 2020; Andrade and Fernandes, 2021; Aplin-Houtz et al., 2021; Čikić and Rajačić, 2021; Martucci, 2021), as well as the augmented paid and unpaid workload (Lemos et al., 2020; Andrade and Fernandes, 2021) increased work-family conflict (Andrade and Fernandes, 2021; Aplin-Houtz et al., 2021). Furthermore, this combination of variables also led to higher parental dissatisfaction (Lemos et al., 2020). Additionally, having to assist their children with online learning-related issues was a widely mentioned challenge by women (Aplin-Houtz et al., 2021).

Accordingly, the same gendered scenario observed among couples was observed for parents. Compared to fathers, mothers declared to spend more time on unpaid workload and less time on paid workload (Stefanova et al., 2021). For mothers, the more time they spend nurturing their children, the poorer their self-efficacy and career aspirations levels would be (Stefanova et al., 2021). The same link was not found for fathers or individuals without children (Stefanova et al., 2021). Thus, the higher the negative coparenting, the higher were mothers’ family-to-work conflict and mother’s depressive symptoms (Zou et al., 2021). Fathers did not perceive the same reality (Zou et al., 2021). Mothers also noted that fathers were more involved in unpaid work regarding childcare, even though they adopted the “assistant” role or had poor performance (Lemos et al., 2020; Çoban, 2021). Again, this cluster pushed mothers and fathers to perform traditional gendered roles (Çoban, 2021; Martucci, 2021). Interestingly, even though supportive coparenting blocked the father’s work-to-family conflict from exacerbating their depressive symptoms, negative coparenting moderated the relationship between mothers’ work-family conflict (in both directions) on fathers’ depressive symptoms (Zou et al., 2021). Hence, compared to fathers, mothers report augmented levels of work-to-family and family-to-work conflict (Stefanova et al., 2021).

Mothers’ work-to-family conflict (Aplin-Houtz et al., 2021) was coupled with feelings of guilt for not enjoying more time with their children. These feelings of guilt were also coupled with family-to-work conflict for not complying with job expectations (Aplin-Houtz et al., 2021; Martucci, 2021). As a result, working mothers were preoccupied that the demands of their mothers’ role could have a turnaround in their career regarding workload (Aplin-Houtz et al., 2021) and job position (Çoban, 2021).

In spite of all the variables mentioned earlier fuelling work-family conflict, physical closeness and more quality time with their children lessened work-family conflict for working mothers (Čikić and Rajačić, 2021; Çoban, 2021). This scenario was also the case for the mothers who were not living with the father of their children (Adisa et al., 2021). Indeed, working mothers are still keen on continuing to work from home to be closer to their children, as long as telecommuting is not imposed (Lemos et al., 2020; Adisa et al., 2021; Çoban, 2021).

### Work-family enrichment

#### Well-being, gender, and work-family enrichment amid the COVID-19 outbreak

Experiencing work-to-family enrichment during the coronavirus pandemic increased the perception of positive affect and decreased the perception of negative affect (Çetin et al., 2021). Nonetheless, experiencing family-to-work enrichment increased only positive affect (Çetin et al., 2021). Additionally, spending more time at home faded the relationship between family-to-work enrichment and positive affect (Çetin et al., 2021).

#### Job resources, job demands, gender, and work-family enrichment amid the COVID-19 outbreak

Only one study tracked the evolution of work-family enrichment before and during the COVID-19 outbreak (Vaziri et al., 2020). Although a profound outlook regarding Vaziri et al. (2020) main findings will be discussed afterwards, roughly only a tiny percentage of individuals reported work-family enrichment fluctuations. If one was to have altered their levels of work-family enrichment during the COVID-19 pandemic, the higher odds were going from low to medium scores of work-family enrichment (Vaziri et al., 2020). The second most common switch resulted in diminished work-family enrichment levels, from high to low scores or medium to low scores (Vaziri et al., 2020). Finally, the least common transition in work-family enrichment was from high to medium levels of work-family enrichment (Vaziri et al., 2020).

Perceiving lower levels of work-family enrichment was coupled with lower job satisfaction and performance and increased turnover intentions (Vaziri et al., 2020). Conversely, experiencing higher levels of work-family enrichment was related to more job satisfaction and commitment and lower turnover intent (Vaziri et al., 2020).

#### Co-occurrence of work-family conflict as well as enrichment and gender amid the COVID-19 outbreak

Vaziri et al. (2020), aiming to trace transitions in work-family bidirectional profiles (conflict and enrichment) from before the COVID-19 crisis onset (February 2002) and during the COVID-19 crisis (April 2020), conducted a quasi-experimental design study. Study 1 (pre-pandemic) showed a total of three profiles–beneficial (low conflict and high enrichment), active (medium conflict and enrichment), and passive (low conflict and enrichment) (Vaziri et al., 2020). In the first study, 59% of participants fit the beneficial profile, while this percentage dropped to 54% during the second study (Vaziri et al., 2020). Additionally, study 1 showed that 22% of workers were classified in the active profile, whereas this percentage slightly dropped to 15% during study 2 (Vaziri et al., 2020). Finally, 19% the passive profile represented 19% of the pre-COVID-19 sample and 41% of the sample during the COVID-19 outbreak (Vaziri et al., 2020). In other words, employees were prone to remain in their pre-pandemic profiles, though positive and negative changes in both work-family conflict and work-family enrichment occurred (Vaziri et al., 2020).

The most likely transition–from passive to active profiles–means participants experienced both more work-family enrichment and work-family conflict (Vaziri et al., 2020). The second most likely transition (from active/beneficial to passive profile) means experiencing lessened work-family enrichment and maintaining low work-family conflict or experiencing flat levels of work-family enrichment and work-family conflict (Vaziri et al., 2020). The latter scenario seems disruptive to the work and family systems (Vaziri et al., 2020). Additionally, both types of negative transitions were related to lower family-supportive supervisor behaviours (Vaziri et al., 2020). Further, a negative switch from beneficial to active was linked with emotion-focused coping, technology overload, technology invasion, and leaders showing diminished compassionate behaviour (Vaziri et al., 2020). Moreover, a negative transition from beneficial to passive had a greater chance of occurring for individuals who felt technology incompatibility (Vaziri et al., 2020). Finally, all negative transitions were coupled with lessened job satisfaction and performance and greater turnover intentions (Vaziri et al., 2020).

Gender did not impact the profile transitions (Vaziri et al., 2020).

### Work-family balance

#### Paid workload, unpaid workload, and work-family balance in the course of the COVID-19 crisis

Following a similar fashion to work-family conflict consequences, several findings point out that working from home during the COVID-19 crisis resulted in aggravated role conflict due to intensified paid and unpaid workload. This scenario was linked to a downgraded work-family balance (Wang et al., 2020). Nevertheless, work-family balance was higher for telecommuters than on-site workers during the COVID-19 outbreak (Kaufman and Taniguchi, 2021).

#### Well-being and work-family balance on the course of the COVID-19 crisis

Enforced remote work meant irregular wake-up times, boredom, change in work-sleep-leisure time ratio, and inability to visit loved ones and attend religious rituals (Kolo et al., 2021). These resulted in lower work-family balance (Kolo et al., 2021). Meanwhile, during the COVID-19 crisis, the work-family balance was a key variable for well-being since it lessened burnout and boosted well-being (Carvalho et al., 2021).

#### Job resources, job demands, gender, and work-family balance throughout the COVID-19 crisis

Job autonomy (Wang et al., 2020) and job satisfaction (Rai et al., 2021) were underpinned as paramount to work-family balance during the COVID-19 outbreak (Wang et al., 2020). Work-family balance and job satisfaction were mediated by marital age for men; although, for women, work-family balance was directly related to job satisfaction (Rai et al., 2021). These results might be in line with the idea that women feared, more than men, that the pandemic would harm their job position (Čikić and Rajačić, 2021; Çoban, 2021).

Evidence is somewhat paradoxical regarding the role of job flexibility on work-family balance during the COVID-19 pandemic. For instance, the novelty of the flexible job arrangement, which typifies remote work, allowed Brazilian working women to discover higher levels of work-family balance (Lemos et al., 2020). Nevertheless, for US-born mothers (Martucci, 2021), job flexibility could have a perverted effect on work-family balance. Job flexibility was only linked to better work-family balance for mothers whose jobs had never before featured flexibility (Martucci, 2021). If that were not the case, both men and women would push mothers to assume the role of looking for children (Martucci, 2021).

#### Couples, gender, and work-family balance on the course of the COVID-19 crisis

Couples were also striving to balance work and family, despite all the hurdles felt (Chung et al., 2020; Kolo et al., 2021). Notwithstanding, double-income working couples hope to be allowed to work from home in the future, if it is not imposed, so they can be closer to their children (Chung et al., 2020).

Indeed, not having the chance to rely on their social support network (e.g., grandparents, house cleaning services, childcare services, educational services) was related to wives experiencing diminished work-family balance (Chung et al., 2020; Çoban, 2021). Being married or cohabiting was no guarantee for sharing unpaid workload (Lemos et al., 2020; Adisa et al., 2021; Çoban, 2021; Kolo et al., 2021). Notwithstanding, the minority of women who felt their spouses were actively sharing the unpaid workload reported higher work-family balance levels than women whose husbands could not be relied on to share the unpaid workload (Lemos et al., 2020).

Regardless of women’s marital status, this widely reported scenario can be further understood by Chung et al. (2020) findings. Among a sample of double-income couples, 43% of individuals matched the profile of high work-family balance, spouse support, and employers’ support; 28% of individuals fitted in the average work-family balance, spouse support, and employers support profile, and 19% of individuals were experiencing poor work-family balance, spouse support and employer support (19%) (Chung et al., 2020). Women were more likely to be in moderate or poor profiles than men (Chung et al., 2020). Once more, another factor unbalancing women’s work and family spheres was that being married did not mean husbands would split the unpaid workload (Lemos et al., 2020). The cluster of flat spouse support, employer support, and work-family balance was also linked to increased marital conflict (Chung et al., 2020). However, the physical closeness between spouses enhances work-family balance (Kolo et al., 2021).

#### Parents, gender, and work-family balance on the course of the COVID-19 crisis

Parents were as well putting much effort into balancing family and work systems, even though assuming the educational role meant reduced work-family balance (Lemos et al., 2020; Adisa et al., 2021; Çoban, 2021; Martucci, 2021).

Despite this, parents found that working from home resulted in more time to play with their children, for both mothers and fathers (Kolo et al., 2021) and for working mothers, whether married or not (Lemos et al., 2020). Once more, the physical closeness and spare time for parenting activities were related to higher parental satisfaction for mothers during the COVID-19 pandemic (Lemos et al., 2020). Interestingly, Lemos et al. (2020) report that a minority of working mothers easily balanced work and family amidst the COVID-19 outbreak. Nevertheless, they hope to be allowed to work from home in the future if it is not imposed (Lemos et al., 2020). Sharing unpaid workload with spouses or other family members (Lemos et al., 2020) seemed to be the reality among mothers who experienced a boost in their work-family balance.

Regarding the three profiles, Chung et al. (2020) found that parents have fewer chances to be among the poor and moderate profiles as children age. Those with poor profiles also had higher levels of parenting stress (Chung et al., 2020).

### Boundary management

#### Enforced blurred boundaries, its management, and gender amidst coronavirus disease

One of the lockdown’s consequences was the involuntary integration of work, family, and other systems (Andrade and Fernandes, 2021; Niu et al., 2021), mainly by the absence of spatial and temporal boundaries (Otonkorpi-Lehtoranta et al., 2021). Thus, individuals had to use strategies to manage the boundary loss (Wang et al., 2020; Johnson and Onwuegbuzie, 2004; Chenji and Raghavendra, 2021; Kashive et al., 2021; Otonkorpi-Lehtoranta et al., 2021).

Kashive et al. (2021), with the goal to cross the boundary fit-perspective (Ammons, 2013) with individual preferences and environmental variables, found four clusters that allow for a better understanding of remote workers during COVID-19. Interestingly, Kashive et al. (2021) clusters were in line with Kossek et al. (2012), Ammons (2013), and Kossek (2016) pre-COVID-19 clusters. The four clusters were boundary-fit family guardians, work warriors, boundary-fit fusion lovers, and dividers (Kashive et al., 2021). Depending on the profiles, the control over work-family boundaries was perceived differently (Kashive et al., 2021). Hence, boundary-fit guardians and work warriors perceived low control over work-family boundaries, while boundary-fit fusion lovers and dividers perceived high control over work-family boundaries (Kashive et al., 2021).

Boundary violations (in both directions) prevented workers from segmenting work and family (Carvalho et al., 2021). Thus, individuals had to find ways to detach work from family (Johnson and Onwuegbuzie, 2004). Boundary-fit fusion lovers applied boundary management tactics more easily than the other three clusters (dividers, boundary-fit family guardians, and work warriors) (Kashive et al., 2021). Likewise, four types of segmentation strategies were applied: temporal (most used one) (Kashive et al., 2021; Rai et al., 2021; Soubelet-Fagoaga et al., 2021), physical (Kashive et al., 2021; Rai et al., 2021), behavioural (Kashive et al., 2021; Rai et al., 2021), and communicative (less used one) (Kashive et al., 2021; Rai et al., 2021).

Comparing boundary management tactics used during the COVID-19 pandemic (Johnson and Onwuegbuzie, 2004) with the tactics found by Kreiner et al. (2009), three new tactics emerged. First, among temporal tactics, during the COVID-19 pandemic, workers would also purposefully disconnect by doing things to take their minds off work (Johnson and Onwuegbuzie, 2004). Another temporal tactic bound to the COVID-19 outbreak was reducing work and family overlap (Chenji and Raghavendra, 2021) by working while family members were not around or were asleep (Johnson and Onwuegbuzie, 2004). Finally, from behavioural tactics, it was found that employees could also emulate office routines, for instance, by recreating the on-site office environment, routines, and overall feeling of going to work (Johnson and Onwuegbuzie, 2004).

#### Well-being and boundary management amidst coronavirus disease

Overall, the hurdles encountered while trying to manage boundaries between work and family negatively impacted one’s role identity and well-being, also leading to feelings of frustration (Andrade and Fernandes, 2021).

Due to the job insecurity felt during the COVID-19 pandemic, prioritising work over the family by applying segmentation strategies had no impact on well-being variables, neither burnout nor flourishing (Carvalho et al., 2021). Nevertheless, amid the COVID-19 lockdown, boundary violations from family-to-work were more likely to occur since physical boundaries were non-existent (Carvalho et al., 2021). At the same time, remote workers needed to prove they had adapted to the abrupt switch (Carvalho et al., 2021). Therefore, segmentation of behaviours from family-to-work solely fostered flourishing (Carvalho et al., 2021).

Women reported high-stress levels once they were not ready to properly switch from on-site to remote work, lacking time to adapt and learn the skills to successfully manage this transition (Aplin-Houtz et al., 2021).

#### Job resources, job demands, gender, and boundary management amidst coronavirus disease

Kerman et al. (2021) study design included a two-wave data collection, in which the first wave was gathered prior to the COVID-19, and the second wave was gathered amid the COVID-19 crisis. Their findings demonstrated that work-boundary violations lessened solely work-related satisfaction during the coronavirus disease, whereas home-boundary violations mitigated only home-related satisfaction (Kerman et al., 2021). During the coronavirus disease, work boundary violations decreased home-related satisfaction more for women without children (Kerman et al., 2021). During the COVID-19 outbreak, boundary violations in one system (e.g., work) not only generated unfinished tasks in the opposite system (e.g., home) but also in the domain they first occurred (e.g., work). Thus, dissatisfaction with the system boundary violations that had first occurred (e.g., work) increased (Kerman et al., 2021).

Among the clusters found by Kashive et al. (2021), boundary-fit family guardians and boundary fit-fusion lovers were the most permeable to supervisor support, organisation policies, and perceived control over when, how, and where their work will be done (Kashive et al., 2021).

#### Couples, parents, gender, and boundary management amidst coronavirus disease

Overall, the factors which played a furcal role in how families coped with blurred boundaries were the adaptation to telework and work-family management before the COVID-19 pandemic (Kashive et al., 2021).

The involuntary integration between work and family increased mothers’ struggles to switch from their worker roles (Andrade and Fernandes, 2021; Otonkorpi-Lehtoranta et al., 2021). On the one hand, childcare negatively impacted paid workload more intensely for mothers than for fathers (Otonkorpi-Lehtoranta et al., 2021). On the other hand, while gender was found to moderate the relationship between boundary violations and segmentation behaviour from work-to-family, gender did not moderate the relationship between boundary violations from family-to-work and segmentation behaviour from family-to-work (Carvalho et al., 2021).

Work-to-family boundary violations substantially impacted women’s effort to segment work and family during the COVID-19 outbreak (Carvalho et al., 2021). In other words, women struggled to prevent work from invading their family life (Carvalho et al., 2021). The partial moderator role of gender could also be linked with the fact that women are more likely to belong to the boundary-fit family guardians’ group (Kashive et al., 2021). Consequently, women place family identity on a higher stand and perceive low control over work-family boundaries (Kashive et al., 2021). Further, these antagonistic findings could also be related to the idea that, especially in families where the unpaid workload was gendered before the COVID-19 outbreak, mothers took the bulk of childcare (Otonkorpi-Lehtoranta et al., 2021). Hence, for these mothers, the boundary absence combined with increased unpaid workload boosted role strain during the lockdown (Andrade and Petiz Lousã, 2021; Otonkorpi-Lehtoranta et al., 2021), making it challenging to apply segmentation strategies (Waismel-Manor et al., 2021), especially from family-to-work (Otonkorpi-Lehtoranta et al., 2021).

Regarding mothers’ behavioural segmentation boundaries, the tactics included reaching out to spouses (Chung et al., 2020). Nevertheless, in Andrade and Fernandes (2021) Brazilian sample, all working women reported their husbands were unavailable sources of support to help them segment work and family. Although in other studies, women relied on their spouses (Lemos et al., 2020; Kolo et al., 2021), being married did not necessarily mean easing in managing blurred boundaries due to the father often adopting the “assistant role” or having a poor performance (Lemos et al., 2020). Additionally, some mothers felt that children (Aplin-Houtz et al., 2021) or the whole family (Çoban, 2021) did not comply with the physical boundaries. However, their husbands’ physical boundaries were not crossed, not even by children (Waismel-Manor et al., 2021).

Concerning physical boundaries (e.g., rooms at home), at the parental system level, the stress stemmed from mothers’ feeling that their partners could not understand at what cost physical boundaries were being managed (Otonkorpi-Lehtoranta et al., 2021). The tension in the parental subsystem did spill into the couple’s subsystem (Otonkorpi-Lehtoranta et al., 2021). Regarding the case of dual-earners, women also reported increased difficulties in segmenting work and family (Çoban, 2021; Otonkorpi-Lehtoranta et al., 2021; Waismel-Manor et al., 2021). It looks like gender traditional roles pushed women to be the “nomad” and men to have a designated room to work from home (Çoban, 2021; Leroy et al., 2021; Otonkorpi-Lehtoranta et al., 2021; Waismel-Manor et al., 2021). Indeed, couples stated that one of the significant lockdown challenges was lacking a proper workplace room at home (Kolo et al., 2021). Therefore, women were forced to integrate work and family systems, while men were allowed to apply segmentation strategies and quickly switch to integration strategies when desired (Çoban, 2021; Otonkorpi-Lehtoranta et al., 2021; Waismel-Manor et al., 2021). Women and men also reacted differently regarding the infiltration of workspace into the home through videoconferencing (Waismel-Manor et al., 2021). Thus, women were actively preoccupied with looking professional (e.g., wearing make-up) and avoiding family from invading their cameras (e.g., tidying up the surroundings from “family or children vulnerabilities”). Regardless, men did not fear that “children or domestic vulnerabilities” would make their image look less professional (Waismel-Manor et al., 2021). In line with Johnson and Onwuegbuzie (2004) findings, Waismel-Manor et al. (2021) argued that women’s usage of their body and surroundings are segmentation tactics specifically coined for telecommuting during the COVID-19 pandemic. Hence, they are different from the ones found by Kreiner et al. (2009).

The same gendered pattern was observed in the temporal tactics and how dual-earners divided time (Otonkorpi-Lehtoranta et al., 2021; Waismel-Manor et al., 2021). Thus, men would work as many hours as desired without dealing with boundary violations from children and were able to decide when to take breaks. In contrast, women were prevented from working continuously, were forced to arrange their working schedules per their husbands’, and felt no control over time management (Waismel-Manor et al., 2021). Other women had to work in the late evenings because that was the time their husbands were available to look after their children (Waismel-Manor et al., 2021). Among couples partnered, mothers were the ones actively asking for temporal tactics to be rearranged (Otonkorpi-Lehtoranta et al., 2021). Some couples managed temporal boundaries by working shifts (Otonkorpi-Lehtoranta et al., 2021). Other couples arranged their schedules considering their partners’ working hours, house chores, childcare (Kolo et al., 2021), and the children’s online school activities (Otonkorpi-Lehtoranta et al., 2021). Additionally, some couples reported working 7 days a week (Otonkorpi-Lehtoranta et al., 2021). Three scenarios were found for families whose parents started working 7 days a week. One consisted of mothers bulking up with the paid and unpaid workload while fathers prioritised their paid work (Otonkorpi-Lehtoranta et al., 2021). The second was characterised by women feeling that, before the pandemic, the couple was managing paid and unpaid work in a gendered fashion (Otonkorpi-Lehtoranta et al., 2021). In the last scenario, only the mother had switched to remote work during the COVID-19 pandemic (Otonkorpi-Lehtoranta et al., 2021). Hence, mothers were dissatisfied with how temporal tactics were arranged (Otonkorpi-Lehtoranta et al., 2021).

#### Boundary management impact on work-family conflict, work-family enrichment, and work-family balance amidst coronavirus disease

Boundary management was linked to work-family conflict (Vaziri et al., 2020; Adisa et al., 2021; Andrade and Petiz Lousã, 2021; Chenji and Raghavendra, 2021; Kashive et al., 2021; Leroy et al., 2021; Otonkorpi-Lehtoranta et al., 2021; Sedaroglu, 2021), work-family enrichment (Vaziri et al., 2020), family-to-work enrichment (Kashive et al., 2021), work-family balance (Carvalho et al., 2021), work-home balance (Wang et al., 2020), and work-non-work balance (Johnson and Onwuegbuzie, 2004). Since the variables which do not include the family system are beyond the scope of the present systematic review, this subsection will only address work-family variables.

In what concerns the relationship between work-family conflict and boundary management, the absence of boundaries between work and family, combined with the increased unpaid workload, might explain why, for some people, remote work did not reduce work-family conflict (Andrade and Petiz Lousã, 2021; Chenji and Raghavendra, 2021). The unprecedented situation, the hurdles found in segmenting work and family (Sedaroglu, 2021), family-to-work boundary violations (Sedaroglu, 2021), work-based intrusions on family (Leroy et al., 2021), confronting family members who violated boundaries (Sedaroglu, 2021), and the absence of a designated room at home to work (Chenji and Raghavendra, 2021; Zou et al., 2021) were likewise found to be related to work-family conflict. Additionally, it is worth keeping in mind that enforced remote work was also an unprecedented situation for those who were used to working from home prior to the COVID-19 crisis because they worked remotely without having to share physical space with other family members (Sedaroglu, 2021). Therefore, these workers also experienced increased work-family conflict since work-family boundary management was smoother before the lockdown (Sedaroglu, 2021). Furthermore, the work-family conflict did not differ across different boundary management profiles (Kashive et al., 2021).

Regarding the link between work-family conflict and work-family enrichment profiles with boundary management, a positive transition from active profile (medium conflict and enrichment) to beneficial profiles (low conflict and high enrichment) had a higher chance of occurring for integrators compared to segments (Vaziri et al., 2020). Moreover, going through a negative change in profiles (from beneficial to active/passive or from active to passive) was associated with boundary violations from work-to-family through technology (Vaziri et al., 2020). The latter is consistent with the fact that boundary-fit fusion lovers (a cluster in which individuals scored low in technostress, high in environmental variables, and easily applied boundary management tactics) is the cluster with greater levels of family-to-work enrichment (Kashive et al., 2021).

Further, when it comes to work-family balance and boundary management, boundary violations (in both directions) are detrimental to work-family balance (Chung et al., 2020). Moreover, during the COVID-19 pandemic, work-family balance was a key variable promoting flourishing and lowering burnout despite boundary violations (Chung et al., 2020). Gender did not impact the relationship between boundary violations (in both directions) and work-family balance (Chung et al., 2020).

## Discussion and conclusion

To our knowledge, this is the first systematic review of the literature addressing the COVID-19 impact on the work-family interface.

Regarding our first goal (to provide an overall view of the body of evidence regarding the COVID-19 impact on the work-family interface), it is possible to state that the COVID-19 crisis has impacted the work-family interface. The COVID-19 crisis has affected the work-family interface mainly due to the need to promptly adapt to remote work, greater unpaid and paid workload, and higher role strain.

Regarding our second goal [to map and compare the COVID-19 effects on the work-family interface, the family system, and the family subsystems (marital and parental subsystems)], the findings consistently show that the COVID-19 outbreak has pushed men and women to perform traditional roles. Hence, the COVID-19 pandemic had a negative impact on women and the marital and parental subsystem. In the marital subsystem, the couples’ work-family interface management before COVID-19 was crucial in promoting an efficient management of both systems. Further, parents struggled with the educator role in the parental subsystem. Nevertheless, parents wish to be able to continue working from home, as long as it is possible to reach out to their social support network.

Concerning the remaining goals (to understand how COVID-19 influenced work-family conflict, work-family enrichment, work-family balance, and boundary management), COVID-19 had a complex effect on work-family conflict. Indeed, some variables aggravated work-family conflict (e.g., imposed remote work), and others diminished it (e.g., organisational, supervisor, co-workers, and family support). The same unclear scenario was found for work-family balance, with some factors mitigating it (e.g., remote work) and others augmenting it (the novelty of flexible schedules). Regarding work-family enrichment, fluctuations were rare, despite the most common one meaning improved levels of work-family enrichment. Finally, when it comes to boundary management, due to the COVID-19 measures, individuals had to create specific boundary management tactics, and both segments, as well as integrators, benefited from organisational guidelines focused on disconnection.

We believe the present synthesis sheds light on new research avenues. For example, of the thirty-two included studies, only one adopted a dyadic analysis. To better understand how dual- 1462 earner couples manage work when both spouses work from home, it could be interesting to cross individuals with dyadic analysis using a mixed or a qualitative design. Also, from the thirty-two studies, no sample included the offspring. We suppose that by doing so, it will be possible to address a literature gap regarding the impact of remote work on parenting from the offspring’s perspective.

Similarly, it seems urgent to include employers’ and supervisors’ points of view when gathering data regarding family-friendly practices for remote work. Another literature gap we have stumbled upon is that, among the thirty-two extracted studies, no study had focused on the experiences of LGBT couples. We believe it is paramount to encourage researchers to measure and analyse work-to-family and family-to-work as two distinct variables. Further, it would be interesting if more research would integrate the several work-family interface variables instead of focusing only on one of the constructs.

We consider the present systematic review also could guide psychotherapists to acknowledge the need to work on the division of paid and unpaid workload and boundary management tactics so that the burden does not rely only on women’s shoulders. Besides, psychotherapists could help parents make their children respect the imposed boundaries. Finally, it seems utmost for psychotherapists to work with clients on how to activate their social support network when both parents work remotely.

From our point of view, the present systematic review also calls for organisations and supervisors to recognise their role if they allow employees to telecommute. The most striking remark across several studies is that the work arrangements should be an employee’s decision.

The conclusions presented above should be considered cautiously since the present study has several shortcomings. First, the findings must be acknowledged considering not all studies score equally in methodological strength. Furthermore, we included awaiting peer review, and early access articles, which could have impaired the appraisal and synthesis results. Secondly, the inter-rater reliability score was low. Thirdly, when it comes to the definition of the inclusion and exclusion criteria, as it has been already mentioned, we have decided to include borderline cases regarding the professional sector, marital status, and children.

Thirdly, when it comes to the definition of the inclusion and exclusion criteria, as has been already mentioned, we have decided to include borderline cases regarding the professional sector, marital status, and children. Moreover, it must be considered that the thematic analysis has included studies that have not controlled the number of children or the marital status, even though the literature consistently shows these variables should be controlled (e.g., Grzywacz and Marks, 2000). Likewise, the thematic analysis results might have been biased. Finally, the study selection was limited to electronic databases and was not backed by a manual search.

## Data availability statement

The original contributions presented in this study are included in the article/Supplementary material, further inquiries can be directed to the corresponding author.

## Author contributions

BV performed the database searches and data analysis and wrote the first draft of the manuscript. BV and VC conducted the methodological quality assessment. MR and VC critically revised the extracted articles and data analysis, provided feedback, and approved the final manuscript. All authors contributed to the study’s design and the definition of inclusion and exclusion criteria and approved the submitted version.

## Footnotes

1 Henceforward, the expression “unpaid work” will refer to the combination of domestic and childcare work.
