# Supplementary material for: The work-family interface and the COVID-19 pandemic: A systematic review
Source: Front Psychol. 2022 Aug 4;13:914474. doi: 10.3389/fpsyg.2022.914474 (PMC9387637; doi:10.3389/fpsyg.2022.914474)
Supplement: Supplementary file 1 [file Data_Sheet_1.docx]

Supplementary Material

# Appendixes

## Appendix 1 - Search Strategy

### EBSCO

“Work family” OR “family work”

AND

COVID-19

AND

Conflict OR enrichment OR integration OR segmentation OR boundary* OR border* OR balance

- Booleen
- Article, dissertation, expert publications
- December 2019-October 2021
- Spanish, English and Portuguese
- Sources: Academic search complete + MEDLINE + APA PsycArticles + APA PsycInfo + Psychology and Behavioral Sciences Collection + ScienceDirect + ERIC + RCAAP

### WEBOFSCIENCE

(((((ALL=("work family" OR "family work")) AND ALL=(conflict OR enrichment OR segmentation OR integration OR boundar* OR border* OR balance)) AND ALL=(COVID-19)) AND PY=(2019-2021)) AND LA=(English OR Spanish OR Portuguese)) AND DT=(Article OR Early Access OR Proceedings Paper)

- Searched on web of science core collection (Science Citation Index + Social Sciences Citation Index + Arts & Humanities Citation Index + Conference Proceedings Citation Index + Book Citation Index + Emerging Sources Citation Index +)

### Google scholar

- All of words: "Work family" OR "Family work" AND "COVID-19"
- With at least one of the words: conflict OR enrichment OR segmentation OR integration OR boundar* OR border* OR balance
- Date: 2019-2021

### SCOPUS

("Work family" OR "Family work") AND COVID-19 AND (conflict OR enrichment OR segmentation OR integration OR boundar* OR border* OR balance)
