# Supplementary material for: The work-family interface and the COVID-19 pandemic: A systematic review
Source: Front Psychol. 2022 Aug 4;13:914474. doi: 10.3389/fpsyg.2022.914474 (PMC9387637; doi:10.3389/fpsyg.2022.914474)
Supplement: Supplementary file 2 [file Data_Sheet_2.docx]

Supplementary Material

# Appendixes

## Appendix 2 - Included Studies Summary

### Table 2- Summary of Included studies characteristics, goals and main findings

| Author/Date | Country | Type of Sample | Study Design | Study Analysis | Research Goals | Main Findings |
| --- | --- | --- | --- | --- | --- | --- |
| Adisa et al. (2021) | United Kingdom | Single, married and divorced mothers | Cross-sectional and qualitative (semi-structured interview) | Individual (interpretive–constructivist-approach). | Using role theory, examine the impact of the COVID-19 pandemic on women's work-family balance. | For some women, the COVID-19 prevented the possibility of work-family balance and increased perceived role conflict. However, other women felt the lockdown enabled them to rediscover their family members' uniqueness, cherish family values and nurture family members' attachment. Moreover, women felt a more peaceful ambience in their neighbourhoods and communities. |
| Allen et al. (2021) | United States of America | Workers who transitioned to remote work | Longitudinal (5 waves) and mixed-method (questionnaires and open-ended questions) | Individual (multilevel analysis and content analysis) | To understand the relationship between boundary management and work-nonwork balance during the transition to remote work. | Segmentors reported increased work-nonwork balance. Notwithstanding, as time went by, the work-nonwork balance did not increase. Having a home office was associated with a higher work-nonwork balance. There was no difference regarding work-nonwork balance and boundary preferences for those with/out a home office. Temporal, physical, behavioural and communicative strategies were used to manage boundaries. |
| Andrade & Fernandes (2021) | Portugal | Working student mothers (married and divorced) | Cross-sectional and qualitative (focus group) | Individual (thematic analysis) | To grasp how women adjust role boundaries and role reconciliation during the COVID-19 lockdown. | Mothers felt more significant time strain, leading to high work-family conflict levels. To cope with role integration, women relied highly on seeking help from co-workers, university-mates and institutions, but not from spouses or family members. |
| Andrade & Lousã (2021) | Portugal | Working adults | Cross-sectional and quantitative (questionnaires) | Individual (moderation modelling) | To comprehend the link between working after-hours, job autonomy, role overload, work-family conflict and support during the coronavirus pandemic. | Working after hours and having lower job autonomy and role overload predicted work-family conflict. However, supervisor support mitigated work-family conflict for those working after-hours using technology. Likewise, working after-hours technology use and feeling support from co-workers decreased work-family conflict. |
| Aplin-Houtz et al. (2021) | United States of America and Canada | Full-time working mothers (single, married and divorced) | Cross-sectional and qualitative (semi-structured interview) | Individual (content analysis) | To scrutinise how the COVID-19 impacted women regarding perspectives of organisational injustice and work-family conflict. | Women felt their supervisors adopted a more rigid attitude and communicated unclear expectations. Children did not understand imposed physical boundaries. Feelings of guilt arose when participants had to prioritise work over offspring, which led to increased work-family conflict. Participants realised they needed to disconnect from their mother's role to maintain a proper engagement level in their parental role. |
| Asaari & Desa (2021) | Malaysia | Working students | Cross-sectional and quantitative (questionnaires) | Individual (regression analysis and mediation modelling) | To explore the links among work stress, work-family conflict and work performance during the COVID-19 pandemic. | The higher organisational support was, the lower work stress and work-family conflict were. Work performance was enhanced if work stress levels were low and work-to-family conflict. Organisational support would lessen work stress and work-family conflict, which would boost work performance. |
| Barriga Medina et al. (2021) | Ecuador | Full-time workers | Cross-sectional and quantitative (questionnaires) | Individual (structural equation modelling) | To study the work-family conflict effect on burnout dimensions. | Work-family conflict was higher than family-work conflict. Both work-family conflict and family-work conflict worsen burnout levels. Of the three burnout dimensions, emotional exhaustion was the dimension more augmented by work-family conflict and family-work conflict. |
| Carvalho et al. (2021) | Portugal | Teleworkers | Cross-sectional and quantitative (questionnaires) | Individual (mediation moderation modelling) | To evaluate work-family segmentation and work-family balance as pathways to boundary violations and well-being during the lockdown. | Boundary violations prevent work-family balance and are related to lower well-being. Work-to-family segmentation has no impact on well-being, while family-to-work segmentation fosters flourishing. Work-family balance mediated boundary violations from work-to-family and family-to-work and well-being. Segmentation behaviour from family-to-work mediated boundary violations from family-to-work and well-being. |
| Çetin et al.(2021) | Turkey | Full-time employees | Cross-sectional and quantitative (daily questionnaires for five days) | Individual (hierarchical linear modelling for moderator) | To discuss the effects of work-family conflict and work-family enrichment on affect levels during the first phase of the COVID-19 pandemic. | Those who had spent more time at home reported weaker levels of positive affect. Spending more time at home would fade the relationship between family-to-work enrichment and positive affect. Spending more time at home would also boost the relationship between work-to-family conflict and positive and negative affect. |
| Chenji & Raghavendra (2021) | India | Full-time employees | Cross-Sectional and Qualitative Study (Structured Interviews) | Individual (Descriptive Statistics) | To understand respondents' positive and negative experiences  working from home for the first time during the COVID-19 pandemic. | The following factors were linked to work-family conflict during 2020: poorer job performance, workload stressors, unpaid workload and working after hours. The absence of a designated room at home to work was a hurdle when trying to segment. Co-worker support and a flexible working schedule contributed to lower levels of work-family conflict. Work-family conflict was lower for women with a flexible arrangement of unpaid workload. |
| Chung et al.(2020) | Singapore | Double-income married parents | Cross-sectional and quantitative (questionnaires) | Individual (latent profile analysis) | To classify parents' work-family balance and social support profiles and link these profiles with parental stress and marital conflict during the coronavirus outbreak. | It was possible to distinguish three profiles: 1) Strong Work-family balance + strong spouse support + strong employers support (43%); 2) moderate work-family balance + moderate spouse support + moderate employers support (38%); 3) poor work-family balance + poor spouse support + poor employer support (19%). Mothers were more likely to be in the moderate or poor profiles. Those with poor profiles had higher levels of parenting stress and marital conflict. |
| Čikić & Rajačić (2021) | Serbia | Working women | Cross-sectional and mixed-method (semi-structured interviews and online survey) | Individual (content analysis, descriptive statistics and non-parametric tests) | To understand the impact of working from home during a state of emergency on women's work-family conflict. | Comparing during and before the onset of the COVID-19, women who stopped working were the ones experiencing the lowest levels of work-family conflict. Plus, women who continued working on-site experienced the same levels of work-family conflict and women who started working from home reported higher levels of work-family conflict. Regardless of the work arrangement, all women who experienced a work-family conflict intensification linked it to a higher unpaid workload. |
| Çoban (2021) | Turkey | Full-time female employees, married with children | Cross-sectional and qualitative (semi-structured interviews) | Individual (thematic analysis) | To map telework experiences and efforts to balance work and family roles by married middle-class mothers during the COVID-19. | Women reported that their partners were more involved in unpaid work; nevertheless, they adopted the "assistant" role or had a poor performance. In addition, women imposed boundaries and were not respected by family members. Notwithstanding, working mothers are still keen on remote work to feel closer to their children, only if it is their choice. |
| Efi & Parahyanti (2021) | Indonesia | Dual-earner couples who have at least one child | Cross-sectional and quantitative (questionnaires) | Individual (mediator and predictor analysis) | To test core self-evaluation and spouse support effects on workplace well-being through the impact of work-to-family conflict amid the coronavirus disease. | Spouses' support was not linked to workplace well-being among dual earners. A positive core self-evaluation was found to generate greater workplace well-being because this augmented core self-evaluation lowers levels of work-to-family conflict. The work-to-family conflict did not impact the link between spousal support and workplace well-being. |
| Kashive et al. (2021) | India | Full-time workers | Cross-Sectional and quantitative (questionnaires) | Individual (cluster analysis and variance analysis) | To look at the boundary-fit perspective and two  factors, namely, individual preferences (boundary control, family identity, work identity and technology  stress) and environmental factors (job control, supervisor support and organisational policies). | Four distinct clusters were identified: boundary-fit family guardians, work warriors, boundary-fit fusion lovers and dividers. These clusters also differ across at least two significant outcomes: boundary management tactics and positive spillover. The high control cluster profiles like boundary-fit fusion lovers and dividers showed low technostress and higher use of boundary management tactics. Boundary-fit fusion lovers and boundary-fit family guardians having high environmental influencers also showed higher positive family-to-work spillover. |
| Kaufman & Taniguchi (2021) | United States of America | Employed adults | Cross-Sectional and quantitative (data from the PEw Research Centre American) | Individual (logit model) | To understand the impact of working from home during the COVID-19 pandemic on the job experience. | Compared to commuters, telecommuters report that working from home increased job satisfaction, work-family balance, job satisfaction and the number of working hours. However, telecommuters report reduced connectedness to co-workers, job security, and opportunities for advancement. |
| Kerman et al. (2021) | Austria, the Netherlands, Germany and United Kingdom | Employees | Cross-sectional (two waves) and quantitative (daily questionnaires for five days) | Individual (interclass correlations, moderating and mediating model) | To investigate boundary violations and their link to satisfaction, test segmentation preference and unfinished tasks role. | Segmentation preferences did not alter the link between boundary violations and domain satisfaction. Boundary violations in one system generate unfinished tasks in the other system and the domain, which stemmed dissatisfaction in this initial system. Work-related unfinished tasks only mediate work satisfaction and work-related unfinished tasks. |
| Kolo et al. (2021) | Nigeria | Married couples | Cross-sectional and qualitative (in-depth interviews) | Individual (thematic analysis) | To analyse the COVID-19 work-family experiences of working married couples. | Lack of workplace ambience, homeschooling, higher need to clean the house, assistance to children online learning and limited time to work increased work-family conflict. Having more time for spiritual development and more opportunities to spend time with children and spouses represent perceived positive changes. |
| Lemos et al. (2020) | Brazil | Female workers (married, single, with and without children) | Cross-sectional and qualitative (semi-structured interviews) | Individual (content analysis) | To explain the effects of telework during the COVID-19 lockdown period on work-family conflict for working women. | Working from home increased work-family conflict for some women and diminished work-family conflict for other women. This difference could be explained by having no children, spouses sharing unpaid workload, fondness for saving time from commutes, physical nearness from their family and flexible working schedules. |
| Leroy et al. (2021) | United States of America | Employees working from home | Cross-sectional and quantitative (two-waves questionnaires) | Individual (ANOVAs) | To examine how the shift toward intensive work-from-home during the COVID-19 pandemic has impacted the experience of interruptions during work time. | There were increased nonwork intrusions, distractions, and multitasking. Women reported a significant increase in nonwork interruptions, work-based intrusions, multitasking and surprises. Unshared workspace at home was associated with fewer nonwork interruptions, while more nonwork responsibilities predicted more nonwork interruptions. Nonwork interruptions predicted higher family-to-work conflict, emotional exhaustion and lower performance. Work-based interruptions — especially intrusions and multitasking — were associated with higher work-family interference, emotional exhaustion, and lower performance. |
| Martucci (2021) | United States of America | Full-time working mothers (preschool children) | Cross-sectional and qualitative (semi-structured interviews) | Individual (type of analysis was not mentioned) | To document the mother's experiences of work-family balance during the initial COVID-19 lockdown. | Academics were being pushed to be the available parent carrying out unpaid work because they had a flexible schedule prior to the COVID-19 crisis, thus, experiencing work-family conflict. In contrast, remote work has made non-academic mothers' and fathers' work schedules more flexible, increasing work-family balance. |
| Niu et al. (2021) | Japan | General workers | Cross-Sectional and quantitative (questionnaires) | Individual (Mann-Whitney U) | To determine the  health effects of teleworking during an emergency statement as evidence for future policy development. | During the COVID-19, approximately half of the teleworkers were satisfied with their telework, while those with a hybrid work arrangement were less satisfied. The hybrid work arrangement group reported increasing both working hours and meeting hours. Work-family conflict was more pronounced in the telework group than in the hybrid work arrangement or working on-site groups. |
| Otonkorpi-Lehtoranta et al. (2021) | Finland | Both parents work with at least one child under 18 years of age. | Cross-Sectional and mixed-method (questionnaire with scales and open-ended questions) | Individual (thematic analysis) | To examine parents' gendered boundary work practices by asking about the perceived blurring of work-family boundaries and boundary work practices developed to manage work and family roles during the COVID-19 pandemic. | During the COVID-19 pandemic, spatial and temporal boundaries disappeared, and boundary tactics were highly gendered. Also, in families where childcare practices had been gendered before the lockdown, mainly mothers assumed the primary responsibility of increased childcare and struggled to manage their work duties. Therefore, families had multiple means to cope with blurring boundaries based on their ability to change to remote work and work-family practices prior to the pandemic. |
| Rai et al. (2021) | Indonesia | Married employees | Cross-sectional and quantitative (online questionnaire). | Individual (path analysis and multiple regression) | To investigate job satisfaction for married employees by highlighting the family domain and exploring the intervening factors that play significant roles during the pandemic. | The higher were marital age and work-family balance; the more significant was job satisfaction. Job satisfaction was augmented by marital age and work-family balance. Male's marital age and job satisfaction completely mediate work-family balance to job satisfaction. Male’s average working hours partially mediated work-family balance and job satisfaction. Men’s marital age completely me­diated work-family balance to job satisfaction, but not for women. |
| Sedaroglu (2021) | Romania | Employed adults in different working arrangements | Cross-sectional and quantitative (questionnaire) | Individual (Linear regression analysis) | To list the antecedents of work-family conflict during the COVID-19 crisis. | The higher the levels of job insecurity were, the higher work-family conflict was, although fear of the COVID-19 did not enhance this link. Results also showed that working arrangements could decrease (working on-site and hybrid arrangement) or increase (working from home) work-family conflict during the COVID-19 outbreak. |
| Soubelet-Fagoaga et al.(2021) | Spain | Male and females workers | Cross-sectional and mixed research (questionnaire and semi-structured interviews) | Individual (thematic content analysis and ANOVA analysis) | To investigate how workers managed their relationship with work in a context of exceptionality such as a lockdown. | People with dependents experienced higher levels of work-family conflict than those without dependents. Remote workers showed higher levels of family-work conflict than those who worked on-site. Women working from home experienced higher levels of family-work conflict than men telecommuting and people who worked on-site. |
| Stefanova et al.(2021) | United Kingdom, Ireland, United States of America, Spain, Portugal, Poland, the Netherlands, Italy, Germany, Canada, Belgium, Switzerland, Australia, Bulgaria and Romania | Individuals living with a partner, working from home during the pandemic and with or without children | Cross-sectional and quantitative (questionnaires) | Individual (Harman's single-factor test, ANOVA, independent sample t-tests) | To investigate gender imbalance in the division of household duties in families during the COVID-19 pandemic. In addition, to understand if the imbalance was associated with gender differences in individual and career outcomes. | Overall, gender imbalance in the distribution of childcare duties during the COVID-19 pandemic was associated with mothers' adverse personal and professional outcomes. Female caregivers spend significantly less time on work and significantly more time on caregiving than male caregivers during the lockdown. |
| Vaziri et al. (2020) | United States of America | Full-time workers | Quasi-experimental and quantitative (2 wave questionnaires) | Individual (latent profile analysis) | To trace transitions in work-family bidirectional profiles. | A positive change from active to beneficial profiles had a higher chance of occurring for integrators. In contrast, positive changes from passive to beneficial profiles had a greater probability of happening for those whose leaders showed compassion. Negative transitions were coupled with lessened job satisfaction, performance, and higher turnover intentions. |
| Verweij et al. (2021) | The Netherlands | Employed mothers and fathers | Longitudinal and quantitative (questionnaires) | Individual (multilevel regression models) | To compare work-family conflict before the COVID-19 period and during lockdown; to test the link between work-family conflict and parenting. | Overall, the quality of perceived parenting slightly diminished among waves and work-family conflict spillover very little for mothers, while no spillover effect was found for fathers parenting. Mothers who worked longer hours and fathers whose wives worked longer hours were experiencing high levels of Family-to-work conflict and work-to-family conflict and low levels of the parent-child relationship quality during wave 2. |
| Waismel -Mannor et al. (2021) | Israel | Heterosexual working couples living with at least one child | Cross-sectional and qualitative (in-depth interviews) | Individual (grounded theory) | To recognise how heterosexual couples negotiate a workspace and boundary management when both work from home. | The division of physical space, time and bodily-spatial aspects were the main topics which emerged regarding couples' negotiation of workspace. Negotiation of space and time was uncommon for couples, and a gendered pattern emerged. Men quickly managed work-family boundaries using segmentation, while women were forced to integrate work-family boundaries. |
| Wang et al. (2021) | China | Employed adults | Cross-sectional and mixed-method (semi-structured interviews and questionnaires) | Individual (grounded theory and path analytic model) | To conclude, individuals can work efficiently while telecommuting at home during the COVID-19 crisis. | Eight topics were identified regarding telecommuting: work-family conflict, ineffective communication, procrastination, loneliness, social support, job autonomy, monitoring, workload and workers' self-discipline. Social support was linked to lower levels of all remote working challenges (work-family conflict, ineffective communication, procrastination, and loneliness). Job autonomy was negatively related to loneliness. The higher workload and monitoring were, the greater was work-family conflict. Moreover, the workload was also associated with lower procrastination levels. Self-discipline had no impact on the link between monitoring and workload on procrastination. The relationship between social support and loneliness was stronger for self-disciplined workers. |
| Zou et al. (2021) | China | Paired fathers and mothers | Cross-sectional and quantitative study (questionnaire) | Dyadic (actor-partner interdependence model) | To explore the predicting effect of work-family conflict on depressive symptoms of parents who returned to work and their children confined at home during the COVID-19 pandemic. | Parents' work-family conflict was negatively associated with their depressive symptoms. The negative association of maternal family-to-work conflict and depressive father's symptoms was moderated by undermining coparenting. Supportive coparenting moderated the father's work-to-family conflict and the father's depressive symptoms. |
